# 

**Published:** 1952-04

**Authors:** 


### THE BRISTOL MEDICO-CHIRURGICAL JOURNAL

ARMY MEDICAL
MAY 28 1952
LIBRARY

*April 1952*

VOL. 69. No. 250

